# Opioid use in Albuquerque, New Mexico: a needs assessment of recent changes and treatment availability

**DOI:** 10.1186/1940-0640-9-10

**Published:** 2014-06-18

**Authors:** Brenna L Greenfield, Mandy D Owens, David Ley

**Affiliations:** 1 Department of Psychology and Center on Alcoholism, Substance Abuse, and Addictions, University of New Mexico, MSC03 2220, Albuquerque NM 87131-0001, New Mexico; 2 New Mexico Solutions, Albuquerque, New Mexico

**Keywords:** Opioids, Heroin, Buprenorphine, Treatment, Youth, Needs assessment, New Mexico

## Abstract

**Background:**

New Mexico has consistently high rates of drug-induced deaths, and opioid-related treatment admissions have been increasing over the last two decades. Youth in New Mexico are at particular risk: they report higher rates of nonmedical prescription opioid use than those over age 25, are more likely than their national counterparts to have tried heroin, and represent an increasing proportion of heroin overdoses.

**Methods:**

Commissioned by the City of Albuquerque, semistructured interviews were conducted from April to June of 2011 with 24 substance use treatment agencies and eight key stakeholders in Albuquerque to identify recent changes in the treatment-seeking population and gaps in treatment availability. Themes were derived using template analysis and data were analyzed using NVivo 9 software.

**Results:**

Respondents reported a noticeable increase in youth seeking treatment for opioid use and a general increase in nonmedical prescription opioid use. Most noted difficulties with finding buprenorphine providers and a lack of youth services. Additionally, stigma, limited interagency communication and referral, barriers to prescribing buprenorphine, and a lack of funding were noted as preventing opioid users from quickly accessing effective treatment.

**Conclusions:**

Recommendations for addressing these issues include developing youth-specific treatment programs, raising awareness about opioid use among youth, increasing the availability of buprenorphine through provider incentives and education, developing a resource guide for individuals seeking treatment in Albuquerque, and prioritizing interagency communication and referrals.

## Background

Injury mortality rates in the United States have been increasing over the last 20 years. In 2011, almost 80 percent of injury-related deaths were due to unintentional poisoning, of which 90 percent resulted from drug overdoses [1]. Most drug overdoses were related to pharmaceutical drugs and approximately 43 percent were from prescription opioids, which was a rate higher than overdoses from heroin [1]. Unintentional overdose from opioids and problematic opioid use more generally are growing health concerns in the US, which has led professionals to take action and respond to the expanding needs for individuals with opioid use disorders. Individuals and organizations in the medical field have conducted reviews and released educational materials on prescribing opioids for pain [2]. Fewer reviews have been done with professionals outside of the medical field, which could create gaps in how best to address and treat individuals with opioid use disorders.

### Opioid use nationwide

In 2012, 4.9 million people in the United States had used prescription opioids in the past month. Additionally, rates of heroin use have been increasing, with a 50 percent increase in past-month and past-year use of heroin from 2002 to 2011. In total, approximately 0.1 percent of the US population reported recent heroin use, and more than half of individuals who used heroin met criteria for opioid dependence [3]. In 1992, there were 13,761 admissions for nonheroin opioid use, accounting for 0.9 percent of US substance use disorder treatment admissions. By 2010, this number had increased to 166,233 admissions, accounting for 8.7 percent of US substance use disorder treatment admissions [4].

Among those ages 12 to 25 years, opioid use also is on the rise. In 2011, nonmedical opioid drug use had the second highest prevalence rate of any illicit drug, second only to marijuana. Nationwide, 9.8 percent of 18 to 25 year-olds and 5.9 percent of 12 to 17 year-olds reported past-year nonmedical prescription drug use [3]. Thirty-five percent of those admitted to treatment in 2010 for other nonheroin opioid use were 25 years old and younger [4].

### Opioid use in New Mexico

New Mexico has the highest rate of unintentional drug-induced overdose deaths in the country, approximately double the national rate [5], and the proportion of youth overdosing from heroin has increased more than fivefold since 2004 [6]. Between 2004 and 2008, 38 percent and 35 percent of drug-induced deaths in New Mexico were due to heroin and prescription opioids (excluding methadone) respectively, which represented almost three-quarters of all unintentional overdoses in the state [5]. Albuquerque is located in Bernalillo County and is the largest city in New Mexico, with a population of 555,417 as of July 2012. Albuquerque leads the state in the number of unintentional overdoses (largely related to opioid use), highlighting the importance of addressing opioid use in Albuquerque and in the rest of the state [5].

Among individuals entering treatment for substance use disorders in New Mexico between 2006 and 2008, rates of prescription opioid use were highest for those who lived in the Albuquerque metro area as compared to elsewhere in this mostly rural state [7]. Among individuals who were admitted in 2011 to treatment in New Mexico because of heroin or prescription opioid use, approximately 41 percent were female and 30 percent were less than 25 years old [4]. Hispanics and Latinos were disproportionately overrepresented among opioid users: they made up 40 percent of all substance use treatment admissions, but accounted for 71 percent of heroin-related admissions and 63 percent of opioid-related admissions [4].

In 2011, New Mexico high school students were significantly more likely to have tried heroin or injected an illegal drug than high schoolers nationwide [8]. Between 2002 and 2004 in Bernalillo County, individuals between the ages of 18 and 25 had significantly higher rates of past-year illicit use of prescription pain pills than individuals over age 25 [9]. Available data on opioid use in New Mexico underscore the need for additional treatment services for opioid users, particularly for youth. To help inform these efforts, the City of Albuquerque initiated a needs assessment in 2011 to interview providers and administrators at treatment agencies, along with key stakeholders, to inform the allocation of funds for opioid treatment in Albuquerque. This project was implemented in response to an identified community need for opioid services, particularly for adolescents. A vocal community advocacy group had brought attention to the seriousness of this problem. The City of Albuquerque and the State of New Mexico each began to take steps to assess the needs and resources available in the City of Albuquerque and statewide. The City reallocated funds to support the completion of this needs assessment.

### Study aims

This paper presents results from the needs assessment, including issues specific to individuals with opioid use disorders, recent changes in opioid use in the area, gaps in treatment availability, and city-wide factors that affected the treatment of individuals with opioid use disorders. We also discuss the chronology of the needs assessment and subsequent efforts to improve opioid treatment. A copy of the full report is available on the City of Albuquerque website (http://www.cabq.gov/mayor/documents/opioidneedsassessmentnms.pdf/view).

## Methods

### Participants

This study was reviewed by the University of New Mexico’s Institutional Review Board and determined to be exempt from ongoing review. Between April and June of 2011, 39 treatment agencies were contacted and invited to participate in the data collection portion of the needs assessment. Meetings (range of interviewees at each meeting = one to three) were conducted with 24 treatment agencies by phone (n = 6), e-mail (n = 3), or in person (n = 15), for an overall response rate of 62 percent. Five (12.8%) treatment agencies did not provide any response to contact attempts, one (<1%) treatment agency declined to participate, and nine (23.1%) treatment agencies were unavailable due to scheduling conflicts. In addition to the 24 treatment agencies, eight interviews with key stakeholders in Albuquerque were conducted (e.g., Department of Health employees, advocacy groups).

Of the 24 substance use disorder treatment agencies in Albuquerque interviewed for the assessment, one agency provided assessments and vouchers for treatment, two agencies provided medical and psychosocial detoxification services, one agency provided residential treatment, 11 agencies provided counseling, nine agencies prescribed buprenorphine (Suboxone), three agencies provided methadone for opioid agonist treatment, and two agencies offered needle exchange services. These totals reflect only treatment agencies (not individual primary care physicians or buprenorphine providers), and are not exhaustive. Some treatment agencies were counted twice if they provided multiple types of treatment.

### Measure

In-person and phone surveys lasted between 20 and 60 minutes, depending on how much time respondents had and the level of detail of their responses. Data collectors (first and second authors) asked six-open-ended questions that focused on: (a) changes in the opioid-using population in the past 2 years (mid-2009 to mid-2011 to capture recent changes), (b) services that they perceived to be most helpful for this population, (c) services the treatment agency would like to add, (d) limitations the agency was experiencing in treating this population, (e) differences between opioid users and other clients they serve, and (f) gaps in city opioid treatment resources. The eight stakeholders answered only (a), (b), (e), and (f), and these questions were amended based on their specific roles.

### Procedure

The authors compiled a list of treatment agencies in Albuquerque based on their knowledge of available services, online searches for “opioid treatment in Albuquerque”, and two websites (http://findtreatment.samhsa.gov; http://buprenorphine.samhsa.gov). The first and second authors then contacted treatment agencies and scheduled interviews with individuals within the agency who were most involved in the treatment of opioid dependence. At each interview, providers were asked to name other treatment agencies and key stakeholders in the community that should be approached for the needs assessment. Any additional contacts were added to the master list of contacts.

### Data analysis

Completed interviews were transcribed and uploaded into NVivo 9, a qualitative data analysis software package [10]. Using template analysis, interview responses were coded into specific response types under larger categories corresponding to interview questions (e.g., recent changes, gaps in treatment). Template analysis is useful for interview data and utilizes a hierarchical organizational structure in which higher-order codes (in this case, the interview questions) provide the structure under which lower-order codes (here, the specific topics discussed in response to the interview questions) are derived from the data to provide more fine-grained detail about the topic [11]. The first two authors coded the transcripts and coding decisions were discussed to resolve discrepancies. Results are presented in order from most common to least common responses. Because of the open-ended format of the interviews, when a provider did not mention a particular topic, it could have meant either that they did not think it was an issue or that they simply did not think to mention it.

## Results and discussion

### Opioid-specific issues

“Opioid withdrawal is medically like the flu, but psychologically you feel like you are dying”.

### Opioid users versus other substance users

When comparing opioid users to other substance users, interviewees’ comments varied. Five said that the opioid-using population is harder to treat, needs more support, and leaves treatment against staff advice more often. Three commented on the more prominent withdrawal symptoms that are present with opioid use than with some other substances. Two mentioned chronic pain as a characteristic that differentiates opioid users from other substance users. One interviewee in primary care said that opioid users are usually more ready to “deal with their use” than are alcohol users.

Three interviewees highlighted medical issues that coincide with injection drug use. They noted that the number of individuals using heroin intravenously seemed to be holding steady or declining slightly. Medical issues noted included abscesses and blood-borne diseases such as HIV and hepatitis C. One interviewee estimated, “These patients are getting old now. Some have been on methadone since the Vietnam area. Approximately 60 to 90 percent of them are hep[atitis] C positive”.

### Price and its relation to opioid use

In Albuquerque, three interviewees reported that heroin is relatively inexpensive compared to prescription opioids purchased on the streets. Long-time heroin users tend to be poorer, while prescription opioid users tend to come from higher socioeconomic backgrounds. Heroin use is also harder to hide. One interviewee said that, “The heroin population is not working. If you need a fix five to six times a day, it is hard to hold a job. Alcohol-dependent individuals can hold a job”. Among younger individuals and those with chronic pain who previously had opioid prescriptions, interviewees remarked that it was common to initiate use with prescription opioids and then switch to heroin because of its lower price (see Table 1).

**Table 1:** Selected conclusions and recommendations

**Issue**	**Conclusions**	Recommendations*
Opioid-specific issues	Price and its relation to opioid use: Many individuals transition from prescription opioids to heroin due to the costs.	Address the rise in the number of individuals who use prescription opioids before they move on to heroin (see related recommendations below).
Recent changes	More young people are using opioids and seeking treatment.	Increase prevention and awareness efforts targeted at opioid use in young people.
	Nonmedical prescription opioid use has increased.	Providers prescribing prescription opioids should be required to refer to the Board of Pharmacy lists to ensure clients are not “doctor shopping”.
Treatment gaps	Treatment for young people: There are no substance use detoxification facilities for people under 18 years of age.	Develop additional treatment facilities for youth, including detoxification, residential, and outpatient treatment programs.
	Easily available buprenorphine providers: Providers face barriers to prescribing buprenorphine, and it can be difficult for clients to access and afford.	Increase the number of providers who can prescribe buprenorphine and make available an online list of current prescribers.
		Provide incentives for physicians to begin and continue to prescribe buprenorphine.
City-wide concerns	Interagency communication and referrals: Many treatment providers and parents are unsure where to refer clients and youth with opioid use disorders.	Create a resource guide of information about available opioid treatment resources, keep it current, and make it accessible for parents, treatment seekers, and treatment providers.
	Lack of education: Many treatment providers and community members have a limited understanding of opioid use disorders.	Provide educational materials and presentations for providers and parents on effective treatments and overdose prevention strategies (e.g., Narcan).

Notes. *Some issues and gaps have more than one recommendation.

### Heroin use is multigenerational

Three interviewees (all at methadone clinics or needle exchange programs) said that they see generations of families addicted to heroin in Albuquerque. Parents and grandparents often have introduced their children to heroin; interviewees indicated, “Many patients started [using] at 10, 11, or 12 years old. Someone wasn’t taking care of the kids; it is a very deeply ingrained problem”.

### Recent changes

“We are sitting on a new epidemic of young people. They start out with pills and then switch to heroin, and it is a struggle to get them into therapy”.

### More young people are using opioids and seeking treatment

Many interviewees (n = 13) said that the opioid-using population has gotten younger in the past 2 years. More young people appeared to be seeking treatment for opioid use or were encouraged to seek treatment by family members; these changes were reported across treatment types, including primary care, the VA Health Care System, substance use disorder treatment facilities, and methadone clinics. This increase is coupled with a lack of treatment options within Albuquerque for individuals under 18 (see conclusions and recommendations in Table 1), leading to a noticeable increase in calls to treatment facilities from concerned family members.

In the past 2 years, media attention on heroin use and overdose among youth has increased, and many of these stories have focused largely on teens from middle and upper class backgrounds. One interviewee said that, “The higher SES [socioeconomic status] groups are affected; that is why there is now the so-called epidemic”. Opioid use has been an issue in Albuquerque for decades, but it has been affecting more youth across all social classes, which has led to an increase in parents looking for treatment resources for their children, often without success. One interviewee suggested that the momentum generated by concerned parents should be used to catalyze changes in the system of care for opioid users in Albuquerque.

### Nonmedical prescription opioid use has increased

Interviewees (n = 8) repeatedly voiced that the use of prescription opioids has become commonplace. One treatment center reported an increase in the number of individuals using prescription opioids exclusively, while others noted that individuals were using a combination of prescription opioids and heroin. Furthermore, three interviewees mentioned an increase in prescription pain pill use among youth.

Four interviewees mentioned that among opioid users they had treated, most had tried buprenorphine before entering treatment, either through a prescription or illicitly. One said: “Most of these individuals have tried to detox with [buprenorphine] on their own, or they used it because they couldn’t get other opiates”.

### There has been no increase in intravenous heroin use

Clinics serving primarily intravenous heroin users did not report any recent changes: they said they were “just as busy”, or had seen a decrease in the adult population and number of HIV cases (four agencies). In addition, one needle exchange clinic noted that there had been a decrease in the number of individuals accessing their services.

### Relative to alcohol and other drugs, the number of individuals seeking treatment for opioid use has increased

Consistent with state treatment data, four interviewees noted that the number of opioid users seeking treatment had been growing faster than the number of individuals seeking treatment for other substances.

### Smoking heroin has become more common

In line with Drug Enforcement Administration (DEA) reports, two interviewees commented that the quality of heroin is now higher, which allows people to get enough of a high by smoking heroin. Interviewees mentioned that young people may feel more comfortable smoking a substance instead of injecting it, and one primary care provider noted an increase in younger women smoking heroin. One interviewee noted that, “Sixty percent of clients now smoke heroin. The route of administration has changed; it used to be almost all IV”.

### Treatment gaps

“It is a struggle to find consistent counseling, and there are no efforts to increase availability”.

### Treatment for young people

The number of young people seeking treatment for opioid use has increased, and the severe lack of treatment options in Albuquerque for individuals under age 18 has become apparent (see Table 1). The director of one treatment center noted that many families bring their children in for treatment on their 18th birthday due to the lack of resources for those under age 18. Interviewees from three other agencies recounted that they are getting large volumes of calls from concerned parents who want help for their children.

Ten interviewees cited a lack of treatment for youth as a significant issue in Albuquerque. There are several outpatient substance use disorder treatment facilities that offer services for youth, but none are equipped to deal with individuals withdrawing from opioids and many do not accept individuals under age 18. Additionally, the referral system between providers is poor and confusing, and at times youth have been sent inadvertently to programs that do not exist.

Interviewees noted that expanding current substance use disorder treatment services to include younger individuals may not be enough: “It is a struggle to get them in therapy. Buprenorphine was designed for primary care, and young people are not totally there. They want to party with their friends on the weekend, and they figure out they can take buprenorphine during the week and heroin on the weekend”. Another interviewee noted, “Young kids need more supervision. It is hard to keep them in treatment”.

### Accessibility of counseling and medications

Nine interviewees mentioned a lack of available counseling and psychiatric services for general mental health concerns. Interviewees called for more counseling in primary care and increased case management for this population.

When providers have clients taking buprenorphine and wish to refer them to behavioral health treatment, resources are severely limited. Some interviewees who do not currently provide buprenorphine mentioned wanting to start prescribing buprenorphine in-house, possibly to circumvent the difficulty of making referrals. One interviewee said that it had taken her 6 months to find buprenorphine referrals for former clients.

### Day-to-day needs

Five interviewees mentioned that the broad needs (e.g., housing, education) of many opioid users can make it difficult for them to stay in treatment, resulting in “diminishing returns” from treatment. One primary care provider stated that, “I would like to connect clients with programs that help them with life skills and getting a job. People have too much time on their hands”.

### Medical care

Three interviewees said that medical care is not widely available for this population, and it should be incorporated into current services. This was particularly true for those on methadone seeking hepatitis C treatment, which has led one program to provide urgent/primary care medical services on-site.

### Women

Three interviewees said that women with opioid use disorders are an underserved population. One stated, “Residential treatment for women is viewed as extravagant. We need a plan for homeless women with kids. When they relapse, their kids also experience the relapse”.

### City-wide concerns

“By not having a standardized referral system we create gaps in service delivery”.

### Interagency communication and referrals

By far the most notable treatment barrier reported by treatment providers was an inefficient referral system (n = 11; see Table 1). Many of these limitations were related to difficulties in locating a treatment agency that provided the necessary services (i.e., detoxification, youth services, etc.), making referrals to an agency that the individual could afford, referring individuals in immediate need of services to providers without long wait lists, and finding buprenorphine providers who were actively prescribing and could take new patients. These obstacles to making referrals have increased the reluctance of providers to work with this population. One interviewee reported, “Agencies do their own thing because there is too much frustration when trying to communicate between agencies”. Additionally, these multiple hurdles to care may make clients with opioid dependence less likely to succeed. As interviewees commented, “There are too many barriers for low-functioning addicts to get treatment” and “Only the persistent will make it”.

### Funding

Several treatment interviewees (n = 9) reported funding as a limiting factor in providing services. Buprenorphine is difficult for many individuals to afford due to its high costs compared to methadone, and insurance coverage for buprenorphine is rare.

### Stigma

Individuals who use opioids, particularly heroin users, contend with multiple stereotypes that negatively impact the quality of care they can access and receive. Numerous interviewees (n = 9) acknowledged negative perceptions faced by this population, coming from both the recovery community and from the larger society. Clients with opioid use disorders are seen by some providers as “difficult to treat”, which in turn makes providers more reluctant to work with them. Additionally, increases in prescription opioid misuse have made physicians wary of individuals who may be “pill shopping”.

Buprenorphine providers repeatedly expressed hesitance about publicly advertising their services because they anticipated a deluge of individuals looking for buprenorphine and a perceived negative change in the makeup of their waiting rooms. Additionally, one interviewee explained that individuals who tell medical staff about their opioid, methadone, or buprenorphine use often are given poorer care or fewer medical services.

Moreover, there is a philosophical divide between those who support medication-assisted therapy and those who support 12-step-based recovery. Some interviewees reported that some individuals in 12-step-based recovery programs do not believe that individuals taking medication of any kind, including methadone and buprenorphine, are truly abstinent. Individuals with opioid use disorders who choose to use methadone or buprenorphine are less likely to be welcomed by a 12-step group and therefore less likely to reap the benefits of 12-step participation. Non-12-step mutual-help groups such as SMART Recovery may be one alternative, but a recent SMART Recovery group in Albuquerque had limited participation and was discontinued.

One treatment provider reported that individuals who use prescription opioids often do not identify themselves as “addicts” because society does not see them as stereotypical heroin users, and pill use may be seen as “safer”. Those who use prescription opioids may be reluctant to seek treatment because of these societal perceptions, particularly for individuals in middle and upper socioeconomic classes. One treatment interviewee reported that increasing the number of buprenorphine providers in primary care settings would help open treatment to additional segments of this population.

### Prescribing buprenorphine

In order for physicians to prescribe buprenorphine, they must undergo special training, prescribe to a limited number of clients, and keep detailed documentation of their patients for the DEA. Five treatment interviewees perceived these requirements as significant barriers. As one interviewee reported, “The federal government doesn’t make it easy to prescribe”. Complying with onerous requirements from the DEA, coupled with the high demands of this population, has made it difficult to encourage physicians to prescribe buprenorphine, and one interviewee said that, “Only-one third of physicians trained [to do so] actually prescribes buprenorphine”.

### Lack of education

Eight interviewees believed that many providers lack an understanding of opioid use disorders and treatments, and that supplying additional education for providers who work with opioid users would be beneficial. Suggestions included providing physicians with information on how to prescribe other medications in conjunction with methadone and buprenorphine; orienting physicians and nurses to the induction process for buprenorphine and emphasizing the low technicality and burden of the process; mandating prescribers to consult the Board of Pharmacy to verify other prescriptions a client is taking (to help reduce “doctor shopping”); and educating providers that there are effective treatments for opioid use disorders (methadone and buprenorphine) and that prescribing buprenorphine does not have to overtake a clinic’s range of care.

Despite the increase in media attention, eight interviewees reported a need for the education of adolescents, parents, and other community individuals and agencies regarding opioid use. Suggested areas for education included prevention and intervention approaches for adolescents, treatments for opioid overdose (e.g., Narcan), and services available for the treatment of opioid use disorder (e.g., methadone, buprenorphine, needle exchanges, behavioral treatment).

### Study limitations

This report is representative of the opinions of treatment agencies in Albuquerque and some key stakeholders (e.g., coalition members, Department of Health employees). Due to funding and time limitations, we did not solicit the opinions of opioid users who had tried or were trying to navigate the treatment system. Their opinions could have added a valuable perspective to our analyses and enriched our results. Similar future studies should strive to include treatment-seekers when conducting needs assessments.

Self-reports from clinicians were qualitative and may not be exact representations of clients. Additionally, no descriptive data on interviewees were collected, and interviews were conducted in multiple formats (e.g., phone, e-mail, in person.). We did not query specifically about the role of culture and language in relation to treatment-seeking, which may be an important factor in a minority-majority state such as New Mexico. The results from this assessment highlight many concerns that providers in Albuquerque have regarding the treatment of opioid users and should be re-examined with quantitative data. Finally, study findings may not generalize to other parts of the country, but can serve as a starting point for other needs assessments.

### Future directions

Following completion of this needs assessment, the city and state began efforts to make administrative changes where they could to address some of the gaps and disparities in the system. The city has continued working with local advocacy groups and has used the needs assessment to inform their efforts. Additionally, the conclusions and recommendations of the community needs assessment were consistent with an allocation of state treatment funds for a state-run facility in Albuquerque to provide residential treatment for opioid users under age eighteen (http://turquoiselodge.org/adolescent.shtml). In fact, youth treatment needs may have been underestimated because of the lack of treatment facilities in the area.

Table 1 details specific recommendations stemming from the needs assessment and the conclusions they are intended to address. These specific recommendations were based on the authors’ analyses of which items were most pressing to address—and that could potentially have the largest impact. Some possible areas for improving treatment services for opioid users include offering more treatment resources for adolescents, increasing the availability of buprenorphine and naloxone, and developing a centralized intake center to coordinate referrals. Communities across the nation could benefit by conducting similar needs assessments to determine the optimal method for addressing the changing use rates of opioids and other substances.

The expansion of Medicaid eligibility under the Affordable Care Act, as well as changes to parity and the inclusion of substance use disorder benefits under the Essential Benefits package may have a positive long-term effect on the availability and accessibility of opioid treatment services. Unfortunately, it is likely that this impact will take several years to be realized, because, at least in New Mexico, the substance use disorder treatment system has been “siloed” off from the mental health treatment system. Further, the treatment system in New Mexico currently is not organized to support third-party billing and is primarily supported by public funds. As a result, there are many operational, financial, and structural changes that will have to be made before the majority of these organizations can meet the regulatory and credentialing requirements to bill Medicaid or other third-party payors. Thus, the health care changes in the Affordable Care Act provide some hopeful indications for future changes to the fragmented system of substance abuse services in Albuquerque, but it is unlikely that these changes will have immediate impact.

## Conclusions

The increasing rates of opioid use in young people and the rise in prescription opioid use motivated the City of Albuquerque to initiate a needs assessment and use this information to develop new treatment efforts in the community. The results from the meetings with interviewees in Albuquerque indicated that opioid use is increasing among New Mexico youth and the use of prescription opioids is increasing in both adults and adolescents. Two primary recommendations for addressing opioid use in Albuquerque resulted from interviews; these include increasing the availability of buprenorphine and establishing a centralized intake center in Albuquerque to facilitate provider referrals for individuals seeking treatment for opioid use. Beyond treatment services, prevention and regulatory control (e.g., prescription drug monitoring, naloxone availability) also may help to address this issue.

## Competing interests

The authors declare that they have no competing interests.

## Authors’ contributions

DL initiated and oversaw the study. BG, DL, and MO designed study measures and contacted providers for study participation. BG and MO conducted interviews and analyses, reviewed the literature, and wrote the manuscript. DL reviewed and edited the manuscript and all authors approved the final version of the manuscript. BG, DL, and MO responded to requested revisions.
